# Transcatheter Repair of a Sinus Venosus Defect With a Modified Harmony Transcatheter Pulmonary Valve Prosthesis

**DOI:** 10.1016/j.jaccas.2025.103294

**Published:** 2025-02-26

**Authors:** Allison L. Tsao, Stephanie Colello, Matthew J. Gillespie

**Affiliations:** a Department of Medicine, Division of Cardiovascular Medicine, Hospital of the University of Pennsylvania. Philadelphia, Pennsylvania, USA; b Division of Cardiology, The Children’s Hospital of Philadelphia. Philadelphia, Pennsylvania, USA

**Keywords:** Harmony transcatheter pulmonary valve, self-expanding stents, sinus venosus atrial septal defect

## Abstract

Transcatheter repair of sinus venosus atrial septal defect (SVASD) with partial anomalous pulmonary venous return (PAPVR) has become an acceptable surgical alternative for appropriately selected patients. Dilated cardiac anatomy may limit candidacy because of size limitations in balloon-expandable covered stents (BECSs). An 83-year-old man with SVASD with PAPVR presented with decompensated right heart failure secondary to a long-standing left-to-right shunt. Cardiac dimensions precluded the use of a BECS, and we modified a Harmony transcatheter pulmonary valve 22 mm (HTPV22, Medtronic) by removing the leaflets to use the self-expanding frame to cover the SVASD. Following release, the HTPV22 frame was anchored with additional BECSs to secure the frame within the superior vena cava. At 1-month follow-up, there was complete sealing of the SVASD and unobstructed pulmonary venous return to the left atrium. This report describes an innovative modification of the HTPV22 to allow for successful transcatheter repair of a large SVASD.

## Visual Summary

**Figure:**
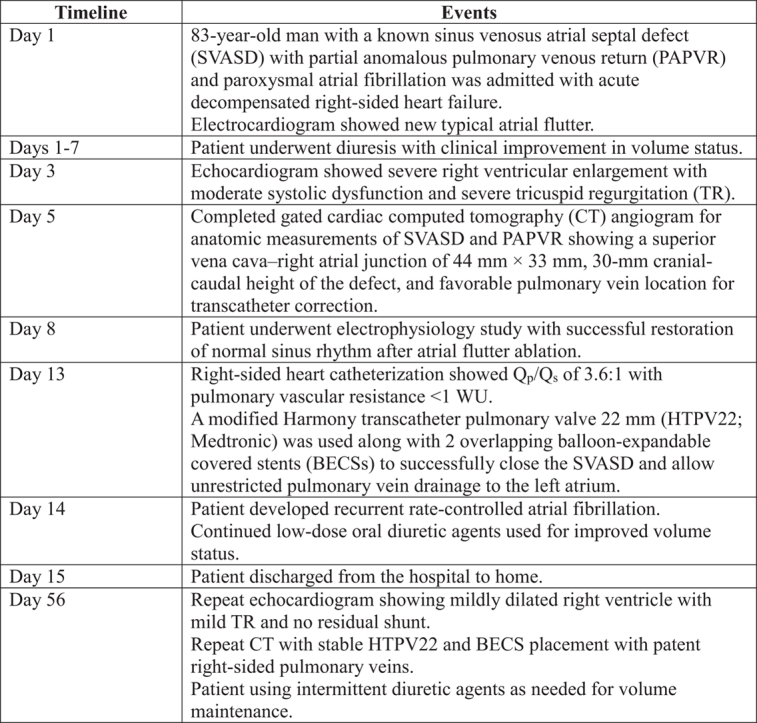

## History of Presentation

The patient was an 83-year-old man with a history of a superior sinus venosus atrial septal defect (SVASD) with partial anomalous pulmonary venous return (PAPVR) that was diagnosed in childhood. He had been followed up throughout his life, and right-sided heart catheterization in 1994 demonstrated a Q_p_/Q_s_ of 2.1:1 with mild to moderate right ventricular (RV) enlargement and excellent exercise tolerance; surgical intervention was serially deferred.

**Take-Home Messages**

• Transcatheter repair of SVASDs with PAPVR is becoming a more prevalent intervention, although patient anatomy may limit candidacy because of size limitations of BECSs.
• Modification of the HTPV22 with leaflet removal allowed for successful transcatheter SVASD with PAPVR repair in a patient with dilated anatomy.

He presented to the clinic with a 2-week history of dyspnea on exertion and weight gain, along with a 1-month history of worsening fatigue. A 12-lead electrocardiogram revealed new onset typical atrial flutter with 2:1 conduction. Physical examination revealed an elevated jugular venous pressure and a distended abdomen with no lower extremity edema. He was referred to the emergency department for further evaluation.

## Past Medical History

The patient had a history of salivary duct carcinoma and had undergone a radical neck dissection followed by adjuvant chemoradiation therapy, which was complicated by facial paralysis, exposure keratopathy, and radiation caries. He had a history of paroxysmal atrial fibrillation. His daily medications included apixaban (5 mg twice daily), amiodarone (200 mg daily), and furosemide (20 mg daily).

## Investigations

He was admitted for management of acute decompensated heart failure. An echocardiogram demonstrated a normal left ventricle size and systolic function with a severely dilated right ventricle with moderately reduced systolic function. There was severe tricuspid regurgitation (TR) with elevated estimated RV systolic pressure to 62 mm Hg and diastolic septal flattening consistent with RV volume overload. He underwent an electrophysiology study, with demonstration of typical atrial flutter that was terminated with a cavotricuspid isthmus ablation; the patient then remained in normal sinus rhythm. Repeat right-sided heart catheterization demonstrated a right atrial (RA) pressure of 23 mm Hg, a pulmonary artery pressure of 64/24 mm Hg, and a pulmonary capillary wedge pressure of 20 mm Hg, with Q_p_/Q_s_ of 3.6:1 and pulmonary vascular resistance of <1 WU.

## Management

**Figure 1:**
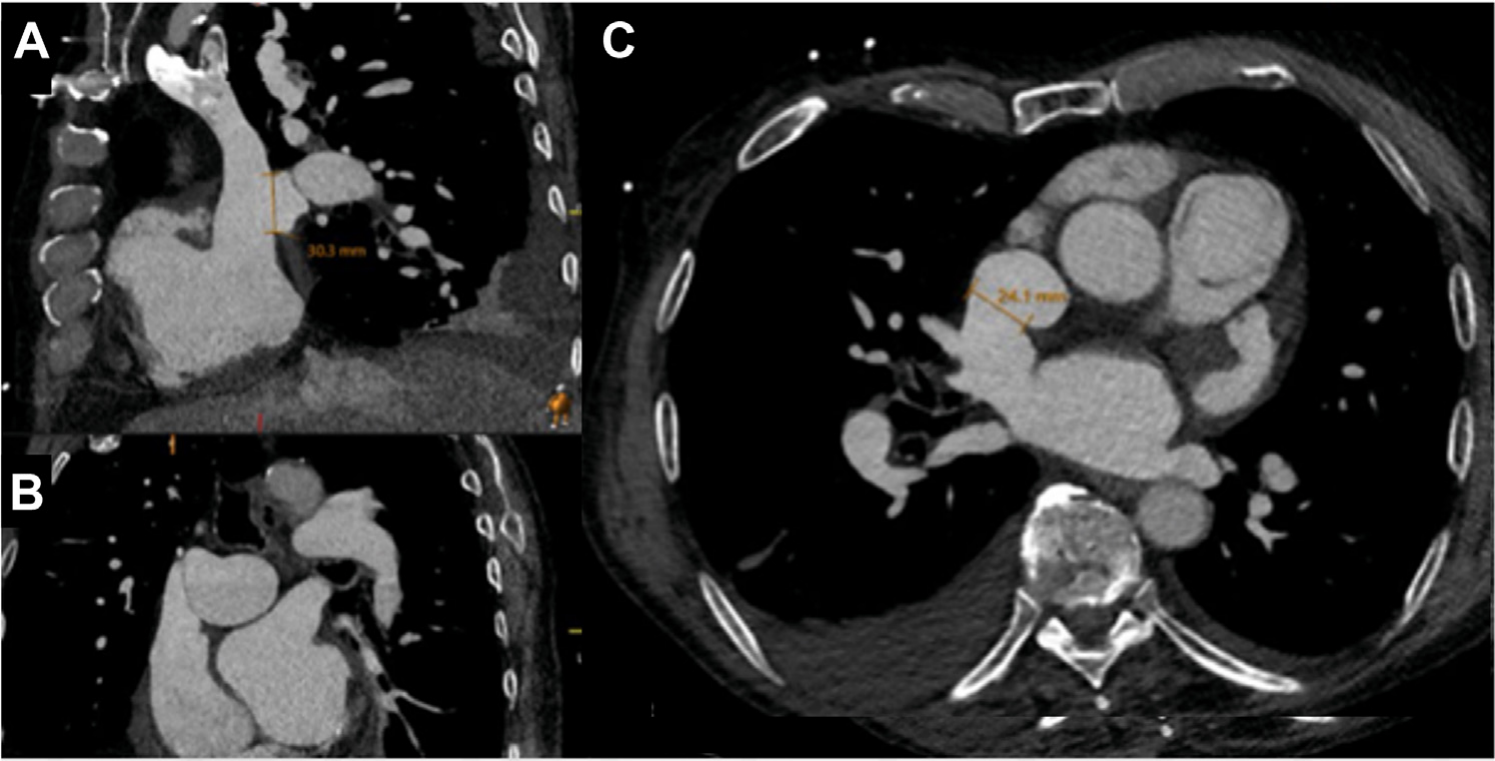
Superior Sinus Venosus Atrial Septal Defect With Partial Anomalous Pulmonary Venous Return Multiplanar (A) sagittal, (B) coronal, and (C) axial computed tomography images of the sinus venosus atrial septal defect with partial anomalous pulmonary venous return of the right upper and middle pulmonary veins.

His active right-sided heart failure, atrial arrhythmias, and frailty made surgery an unappealing option. Therefore, we evaluated him for transcatheter repair of his SVASD with PAPVR. Gated cardiac computed tomography (CT) angiography was performed and showed anomalous drainage of the right upper and middle pulmonary veins to the superior vena cava (SVC) (Figures 1A to 1C). The SVASD measured 30 mm in the cranial-caudal dimension and 24 mm in the lateral dimension. The SVC measured 25 mm × 17 mm above the defect and 44 mm × 33 mm below the defect at the SVC-RA junction (Figures 1A to 1C).

**Figure 2:**
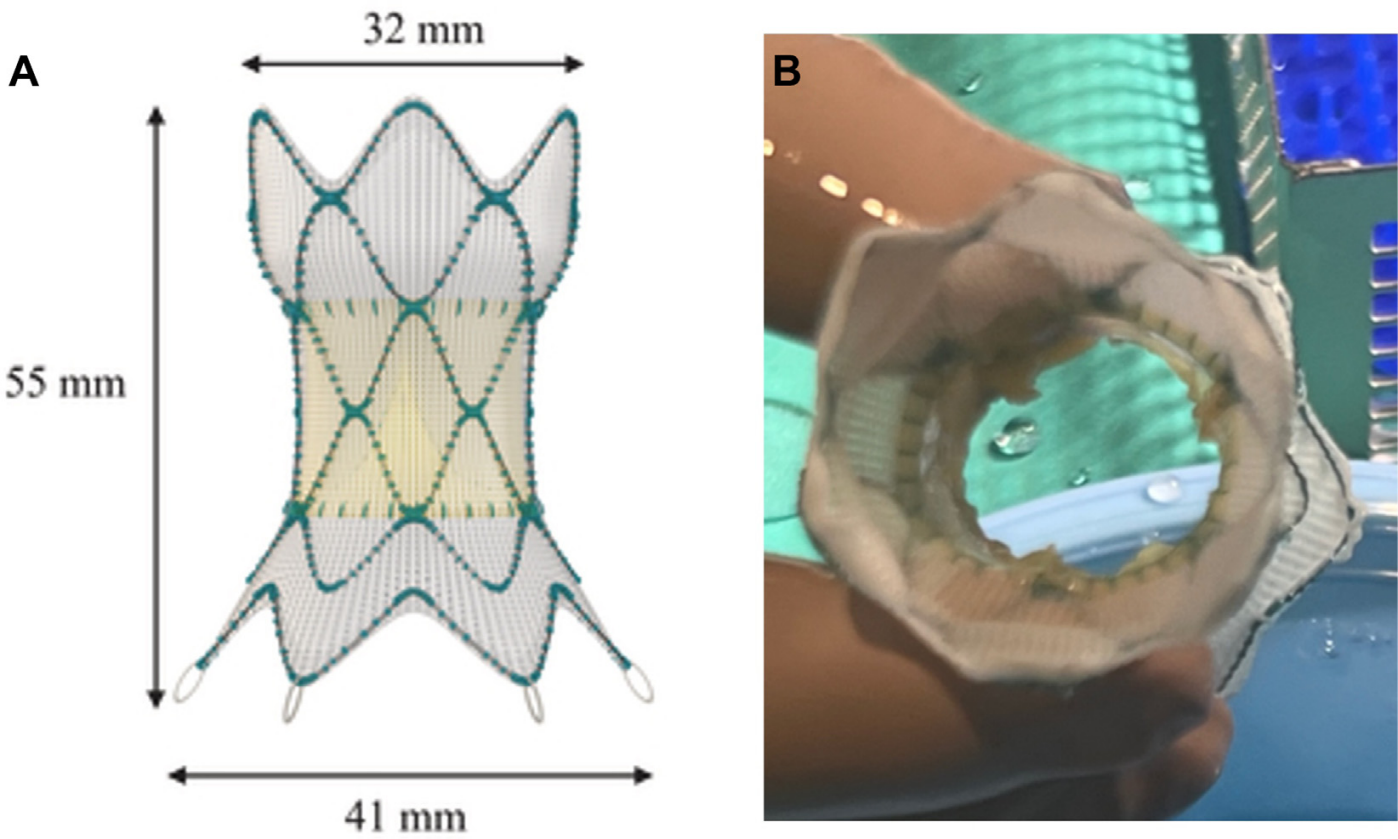
Harmony Transcatheter Pulmonary Valve 22 mm With Leaflet Modification (A) Harmony Transcatheter Pulmonary Valve 22 mm (Medtronic) dimensions. (B) Modified Harmony Transcatheter Pulmonary Valve 22 mm following valve leaflet excision. (A) Adapted from Gillespie MJ, McElhinney DB, Jones TK, et al. 1-year outcomes in a pooled cohort of Harmony transcatheter pulmonary valve clinical trial participants. *JACC Cardiovasc Interv.* 2023;16:1917-1928.

With the dilated SVC-RA dimensions, we did not believe that available balloon-expandable covered stents (BECSs) would give us adequate coverage of the defect even with post-dilation. The Harmony transcatheter pulmonary valve 22 mm (HTPV22) (Medtronic) system is a polyester-covered self-expanding nitinol stent platform that is used for native RV outflow tract interventions. After careful consideration of stent dimensions (Figure 2A) and our patient’s anatomy, we opted to modify a HTPV22 by removing the valve leaflets and using the self-expanding covered stent (SECS) platform, with the intention of placing the HTPV22 outflow (32 mm in diameter) in the SVC and the HTPV22 inflow (41 mm diameter) positioned within the right atrium. This would allow the inflow flare of HTPV22 to seal the SVASD completely and, because it is a relatively soft device, do this atraumatically.

We used transesophageal echocardiography (TEE) and fluoroscopic guidance to proceed with transseptal puncture, and direct left atrial (LA) pressure was 18 mm Hg, with V waves to 28 mm Hg. We used a steerable VersaCross system (Baylis Medical Company) to allow for continuous right upper pulmonary vein pressure monitoring and venography during the case (Video 1). We placed a pigtail catheter in the SVC for initial angiography, thus confirming anatomic landmarks, including the azygos vein and the SVC-RA junction.

**Figure 3:**
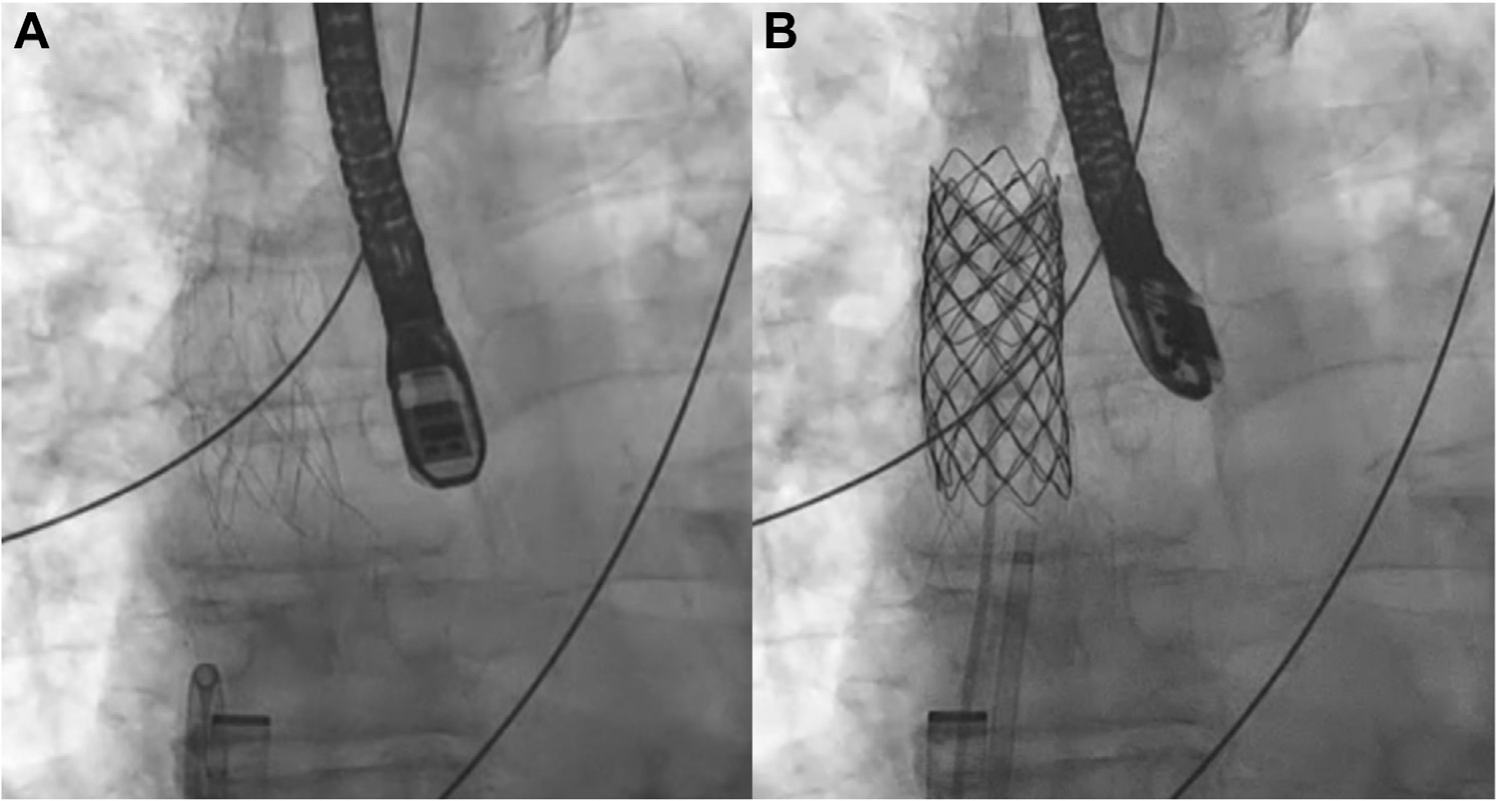
Harmony Transcatheter Pulmonary Valve 22 mm Closure of Sinus Venosus Atrial Septal Defect (A) Fully deployed Harmony Transcatheter Pulmonary Valve 22 mm (Medtronic) frame. (B) Covered stent deployment within the frame to cover the residual leaflets and to anchor the valve within the superior vena cava.

The HTPV22 was prepared on the back table with careful excision of the valve leaflets with a #11 blade and surgical scissors (Figure 2B). The modified HTPV22 was then loaded in standard fashion into the Harmony valve delivery catheter and placed through a 26-F DrySeal sheath (W.L. Gore & Associates) over a double-curve Lunderquist wire (Cook Medical) in the innominate vein. After distal delivery of the nosecone, the distal end of the HTPV22 was unsheathed to allow for SVC contact. Adequate placement just inferior to the azygos vein was confirmed with angiography after partial delivery of the distal end of the HTPV22 (Video 2). The HTPV22 was then deployed in standard fashion (Video 3). Pulmonary vein pressures did not change during device delivery, and there was unobstructed pulmonary venous flow of the now redirected anomalous veins to the left atrium detected by TEE and angiography (Video 4). At this point, we carefully withdrew the SVC pigtail catheter into the right atrium, and we released the proximal end of the HTPV22 (Figure 3A, Video 5).

**Figure 4:**
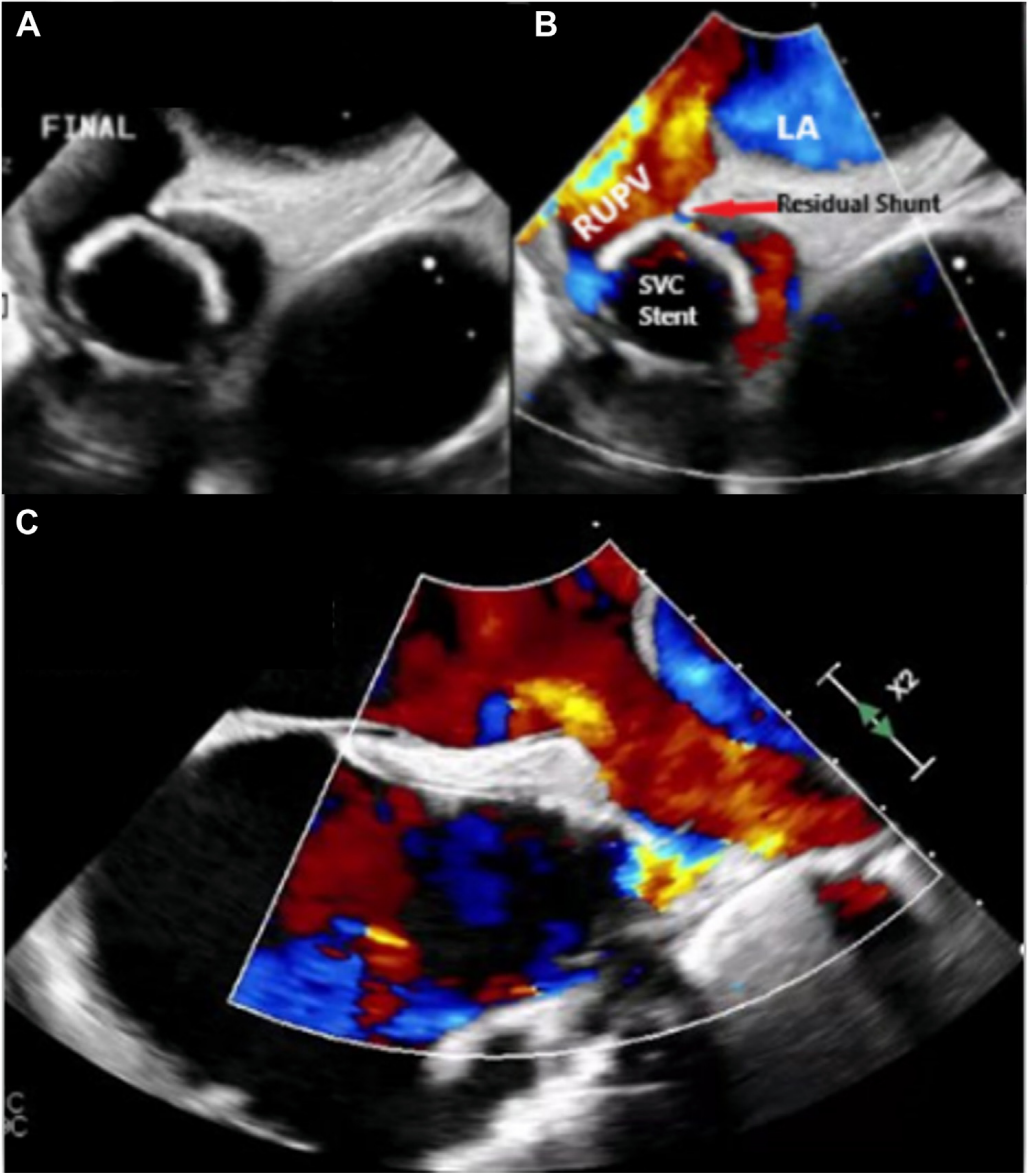
Intraprocedural Transesophageal Echocardiography of Sinus Venosus Atrial Septal Defect With Partial Anomalous Pulmonary Venous Return Closure Successful redirection of the anomalous right-sided pulmonary veins to the left atrium (LA). Stable HTPV22 frame from the SVC into the superior vena cava (SVC)–right atrial junction. RUPV = right upper pulmonary vein.

Following release, there was minimal increase in the right upper pulmonary vein and LA pressures with no significant gradient and laminar flow on TEE. There was slight caudal migration of the HTPV22 several minutes after deployment, and we opted to stabilize the HTPV22 with a BECS. We initially placed a 24 mm × 4 cm Covered CP Stent (NuMED) BECS covering the distal 2 zigs of the HTPV22 extending into the superior SVC. Given the desire to anchor the system fully, we opted to place a second 28 mm × 6 cm BECS that would also allow coverage of the third and fourth zigs of the HTPV22 to maximize device stability and ensure coverage of any residual leaflet tissue (Figure 3B). At this point, there was excellent device stability confirmed by both fluoroscopy and echocardiography, and the inflow flare of HTPV22 filled the SVC-RA junction, effectively covering the SVASD with trivial residual left-to-right flow around the HTPV22 frame into the right atrium (Figure 4B, Video 6). TEE confirmed laminar flow through the redirected pulmonary veins to the left atrium (Figures 4A to 4C).

## Outcome and Follow-Up

**Figure 5:**
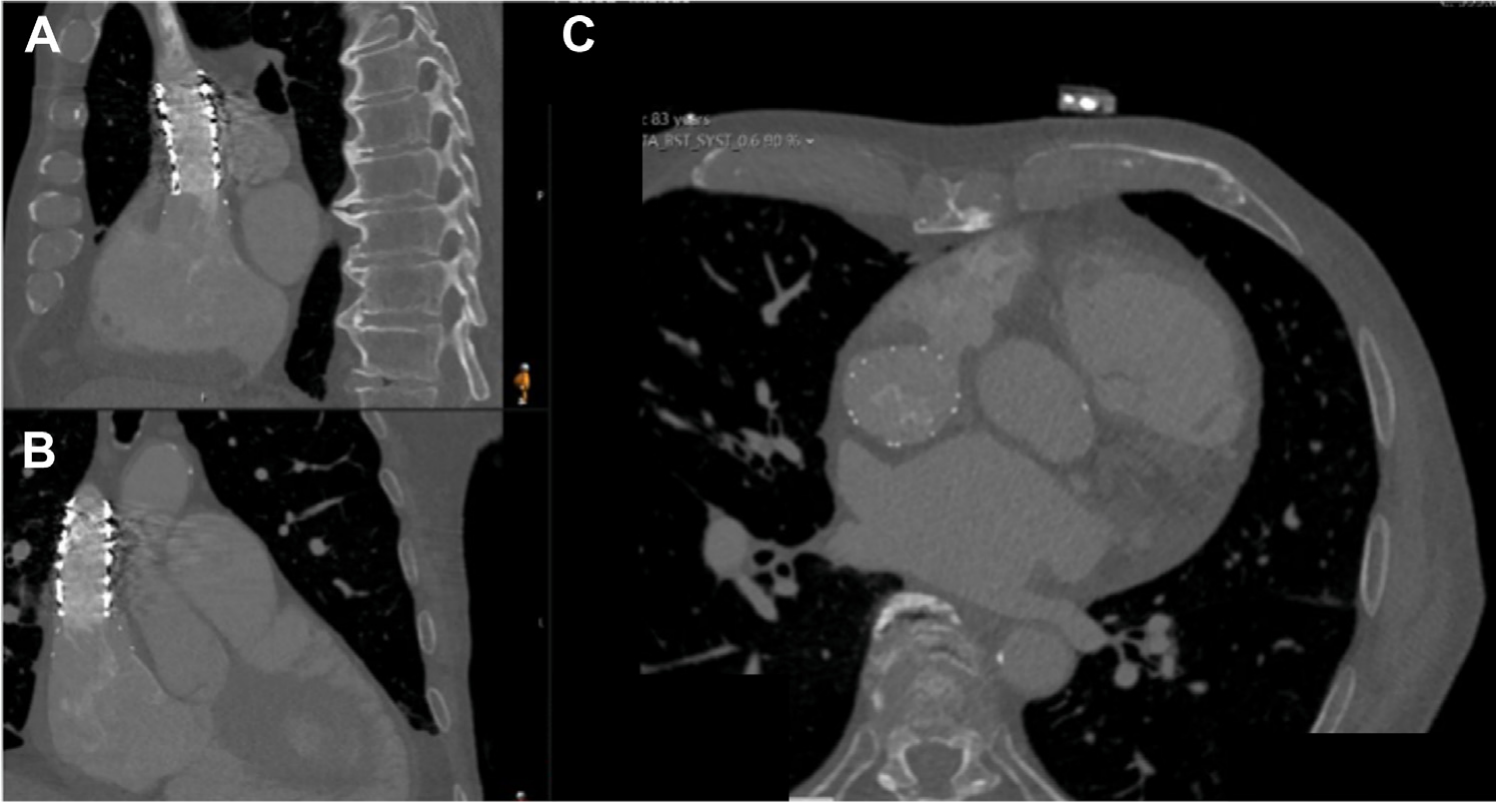
Follow-Up Multiplanar Computed Tomography Imaging Follow up (A) sagittal, (B) coronal, and (C) axial computed tomography images showing a stable Harmony Transcatheter Pulmonary Valve 22 mm (Medtronic) and covered stent placement, with complete occlusion of the sinus venosus atrial septal defect.

**Figure 6:**
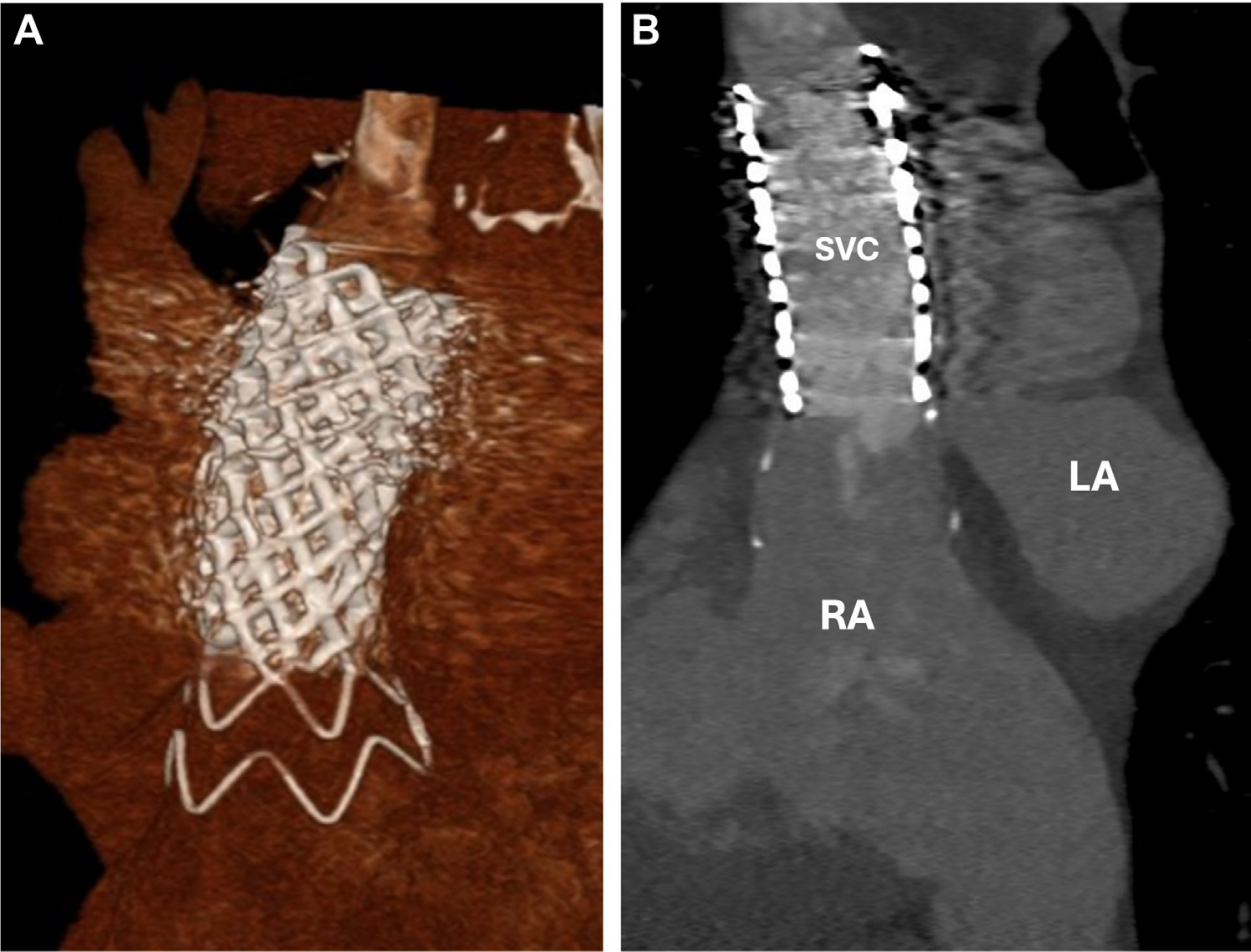
Follow-Up Computed Tomography Imaging (A) A 3-dimensional rendering from follow-up computed tomography of the Harmony Transcatheter Pulmonary Valve 22 mm (Medtronic) frame. The soft “inflow flare” of the valve expands to seal the sinus venosus atrial septal defect. (B) Reconstructed view of the valve frame completely sealing the sinus venosus atrial septal defect. RA = right atrium; other abbreviations as in Figure 4.

Following the procedure, the patient developed recurrent atrial fibrillation with normal ventricular rates but otherwise felt well. He was discharged home 2 days following the procedure. A 6-week follow-up echocardiogram demonstrated a mildly dilated right ventricle with mild TR and an estimated RV systolic pressure of 32 mm Hg, now with a normal inferior vena cava size with respirophasic collapse. Follow-up chest CT and echocardiogram showed stable HTPV22 and covered stent placement with complete occlusion of the SVASD along with patent pulmonary veins (Figures 5A to 5C, Video 7). Three-dimensional reconstruction images demonstrated the HTPV22 and BECS complex fully sealing and repairing the SVASD with PAPVR defect (Figures 6A and 6B, Video 8). The patient has returned to regular activities of daily living and is using intermittent diuretic agents.

## Discussion

Catheter-based closure of SVASD with PAPVR has become more prevalent over the past 10 years after its initial report in 2014.^1^ Patients chosen for a transcatheter approach are typically older patients with more medical comorbidities which may influence the decision to proceed with catheter-based versus surgical repair. Despite this, purpose-built devices for these defects are not commercially available in the United States, and the mainstay of intervention relies on adapting BECSs.^2^, ^3^, ^4^ BECSs are suboptimal in their adaptability to this anatomy given limitations in maximal device expansion, the risk of foreshortening with overexpansion, and the size mismatch between the proximal and distal SVC landing zones. SECSs that allow for adequate sizing and expansion are promising options to overcome these limitations,^5^, ^6^, ^7^ although this approach has been limited, with more readily available vascular endograft SECSs for this purpose.^6^, ^7^, ^8^ Although endograft use allows for appealing size adaptation and sealing, distal zone anchors designed for the thicker-walled arterial system could pose difficulty in the thin-walled venous system.^9^

Our patient’s anatomy, with an anticipated needed length of 50 mm to cover the cranial-caudal dimension and the 44 mm × 33 mm proximal SVC-RA dimension, seemed favorable for the dimensions of an HTPV22 frame, with 55-mm total expended height, 32-mm outflow (distal) diameter, and 41-mm inflow (proximal) diameter. Careful consideration of this technique with adequate distal end anchoring may be considered in other patients with similar dilated anatomy.

## Conclusions

Although this is a promising early experience in the use of a modified HTPV22 with leaflet removal for transcatheter repair of an SVASD with PAPVR, more work is needed with the potential expansion of purpose-built SECS devices for this defect.

## Funding Support and Author Disclosures

Dr Gillespie has served as a consultant and proctor for Medtronic. All other authors have reported that they have no relationships relevant to the contents of this paper to disclose.

## Appendix

Video 1 Initial Pulmonary Venography Biplane angiography of the anomalous drainage of the right upper pulmonary vein via a steerable VersaCross catheter following transseptal puncture

Video 2 Angiography After Distal Deployment Biplane angiography demonstrating Harmony transcatheter pulmonary valve 22 mm (Medtronic) outflow placement in the superior vena cava before full deployment

Video 3 HTPV22 Deployment Deployment of the Harmony transcatheter pulmonary valve 22 mm (HTPV22; Medtronic) in the superior vena cava, covering the sinus venosus atrial septal defect

Video 4 Intraprocedural Pulmonary Vein Angiography Unobstructed anomalous right upper pulmonary vein following HTPV22 deployment

Video 5 HTPV22 Release Release of the proximal end of Harmony transcatheter pulmonary valve 22 mm (HTPV22; Medtronic), flaring into the superior vena cava–right atrial junction

Video 6 Final SVC Angiography Final biplane superior vena cava (SVC) angiography following correction of the sinus venosus atrial septal defect with partial anomalous pulmonary venous return

Video 7 Sagittal Plane CT Computed tomography (CT) scroll through the sagittal plane demonstrating complete sealing of the sinus venosus atrial septal defect by the Harmony transcatheter pulmonary valve 22 mm (Medtronic) frame. The inflow flare fills the superior vena cava–right atrial junction, and the right-sided pulmonary veins drain posteriorly to the left atrium

Video 8 3D Video Rendering A 6-week follow-up computed tomography 3-dimensional (3D) rendering demonstrating Harmony transcatheter pulmonary valve 22 mm (Medtronic) inflow flare sealing the superior vena cava–right atrial junction with patent right-sided pulmonary veins returning to the left atrium posterior to the Harmony transcatheter pulmonary valve–balloon-expandable covered stent complex
